# A Rare Case of Sural Schwannoma With Involvement of the Medial Sural Cutaneous Nerve: A Case Report and Literature Review

**DOI:** 10.7759/cureus.66190

**Published:** 2024-08-05

**Authors:** Kristina Petrova, Lyubomir Gaydarski, Atanas Panev, Boycho Landzhov, R. Shane Tubbs, Georgi P Georgiev

**Affiliations:** 1 Clinical Laboratory, Medical University of Sofia, Sofia, BGR; 2 Department of Anatomy, Histology and Embryology, Medical University of Sofia, Sofia, BGR; 3 Department of Orthopedics and Traumatology, University Multidisciplinary Hospital for Active Treatment Tsaritsa Joanna - ISUL, Medical University of Sofia, Sofia, BGR; 4 Anatomical Sciences, St. George’s University, St. George’s, GRD; 5 Neurosurgery and Structural & Cellular Biology, Tulane University School of Medicine, New Orleans, USA; 6 Neurosurgery and Ochsner Neuroscience Institute, Ochsner Health System, New Orleans, USA; 7 Department of Orthopedics and Traumatology, University Hospital Queen Giovanna - ISUL, Sofia, BGR

**Keywords:** treatment, diagnostics, lower extremity, medial sural cutaneous nerve, sural schwannoma

## Abstract

Schwannomas are benign tumors derived from Schwann cells, typically occurring in the head, neck, and upper extremities, but are less frequent in the lower extremities. They can arise sporadically or from genetic conditions such as neurofibromatosis type 2, associated with *NF2* gene mutations. This report details the case of a 57-year-old female with a two-year history of a painless, slowly growing mass in the posterior aspect of the right proximal cruris. Physical examination revealed a 2 cm, elastic-hard, mobile, non-tender mass with a positive Tinel’s sign. Ultrasound and magnetic resonance imaging suggested a benign nerve sheath tumor characterized by hypoechoic features. The performed surgery revealed that the tumor involved the medial sural cutaneous nerve. Histologic analysis confirmed the diagnosis of schwannoma, showing typical Antoni A and Antoni B regions. Postoperative recovery was uneventful, with no recurrence or neurological deficits at the two-month follow-up. This case demonstrates an unusual localization of a sural schwannoma and highlights the importance of precise physical examination and imaging to diagnose schwannomas accurately. Clinicians should consider schwannoma as a differential diagnosis in patients presenting with slow-growing palpable masses in the lower extremities.

## Introduction

Schwannomas (neurilemomas or neurinoma-schwannoma) are benign tumors that develop from Schwann cells, which are the cells that form the myelin sheath around peripheral nerves [1-3]. Malignant transformation is rare in these tumors [1,4]. These tumors are well-encapsulated with epineurium and can arise anywhere in the body. However, they are most frequently found in the head, neck, and upper limbs, mostly in the brachial plexus [5]. In contrast, schwannomas are less commonly found in the lower limbs, typically affecting the sacral plexus, sciatic, and tibial nerves [1]. Schwannomas can occur sporadically or as part of genetic conditions. An autosomal dominant inheritance pattern has been observed, particularly in conditions such as neurofibromatosis type 2 (NF2). Mutations in the *NF2* gene, which encodes the protein merlin, are often associated with the development of schwannomas. Individuals with NF2 are at a higher risk of developing multiple schwannomas [6]. Some studies have suggested that trauma might play a role in the genesis of schwannomas [7,8]. It is hypothesized that nerve injury could lead to Schwann cell proliferation and potentially contribute to tumor formation [8]. Schwannomas represent 1-3% of peripheral nerve sheath tumors in the whole body and account for approximately 9% of peripheral nerve sheath tumors in the lower limb. The incidence of these tumors in the foot is 2.93% [9]. Schwannomas can also affect the tibial nerve, the common peroneal nerve [10], and the sural nerve (SN) [2]. There is no sex preference, and the condition is most commonly diagnosed in middle-aged individuals (mainly in the fourth and fifth decades of life) [2]. A particularly rare form of schwannoma is the sural schwannoma (SS), which might involve the medial sural cutaneous nerve (MSCN), the lateral sural cutaneous nerve (LSCN), and the SN [2,11]. Unfortunately, no statistical data for the exact incidence of SS is available in the English-language literature. These tumors are usually solitary, asymptomatic, and slow-growing, presenting as a local swelling.

However, some patients may experience confusing symptoms, such as pain, paresthesia, hypoesthesia, and neurologic deficits. Moreover, they are often referred to different medical departments (Orthopedics, General Surgery, Neurology, Neurosurgery). All these factors can lead to a delayed diagnosis [1,12]. Diagnosing schwannomas typically involves magnetic resonance imaging (MRI) to visualize the tumor, followed by postoperative histologic examination to confirm the diagnosis [2,11,13]. Neurologic assessment, nerve sonography, and electromyography (EMG) are also performed [13]. A histologic-specific sign of schwannoma is the alternation of Antoni A and B regions. Antoni A regions are highly cellular, characterized by the proliferation of fusiform cells arranged in interlacing fascicles, demonstrating nuclear palisading and Verocay bodies. Antoni B regions consist of fewer cells, which are disorganized and edematous [1-3,9,11]. The standard treatment for schwannomas is surgical enucleation, which involves carefully removing the tumor from the surrounding nerve tissue [14]. In this report, we present the case of a 57-year-old female with schwannoma of the MSCN for two years. In addition, we present a brief literature review and discuss the diagnosis, imaging, pathological findings, and surgical treatment of schwannoma.

## Case presentation

**Figure 1:**
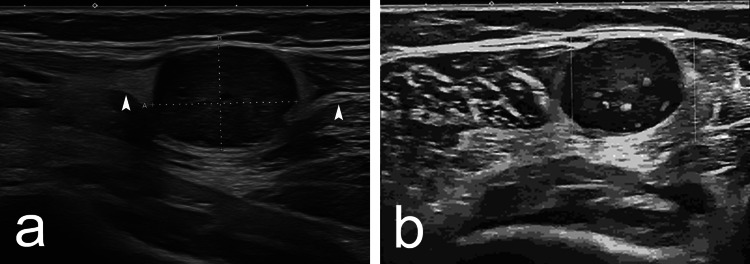
Ultrasound examination presenting a well-defined, hypoechoic, encapsulated mass with internal heterogeneity (a). Doppler ultrasound examination presenting a well-defined, nonvascular, encapsulated structure (b). The white arrowhead shows the medial sural cutaneous nerve.

A 57-year-old female patient was referred to our hospital with a two-year history of a slow-growing, painless mass in the posterolateral aspect of the right proximal posterior leg. The patient only complained of paresthesia in the lateral aspect of the leg, corresponding to the area of innervation of the SN. There was no history of trauma, and there were no comorbidities. The patient denied having a family history of any medical conditions. Physical examination revealed a 2 cm, elastic-hard, mobile, non-tender mass, and during palpation, the pain was provoked in the area of the MSCN with a positive Tinel’s sign. An ultrasound examination of the right popliteal fossa was conducted and revealed a well-defined, encapsulated mass characteristic of a schwannoma. The mass measured 20 × 15 mm and appeared hypoechoic with internal heterogeneity, consistent with typical features of this type of tumor. Doppler imaging demonstrated mild vascularity within the lesion. Overall, the findings suggested a benign nerve sheath tumor (Figure 1).

**Figure 2:**
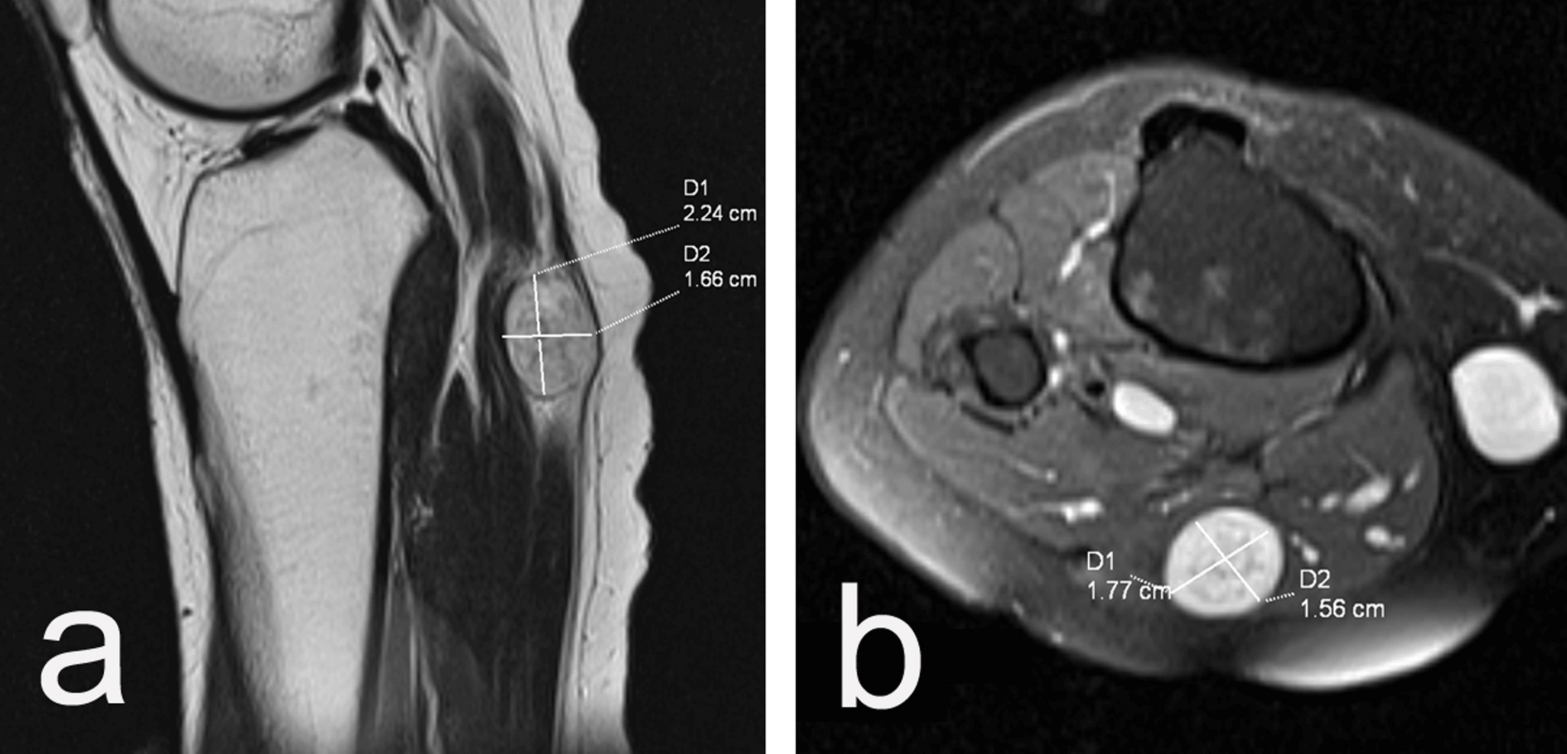
Preoperative MRI showing an oval-shaped subcutaneous mass situated in front of the gastrocnemius muscle (a, b).

A follow-up MRI demonstrated an oval-shaped subcutaneous mass situated in front of the gastrocnemius muscle. The mass measured 16 × 19 mm in the horizontal plane and 22 × 17 mm in the sagittal plane (Figure 2).

**Figure 3:**
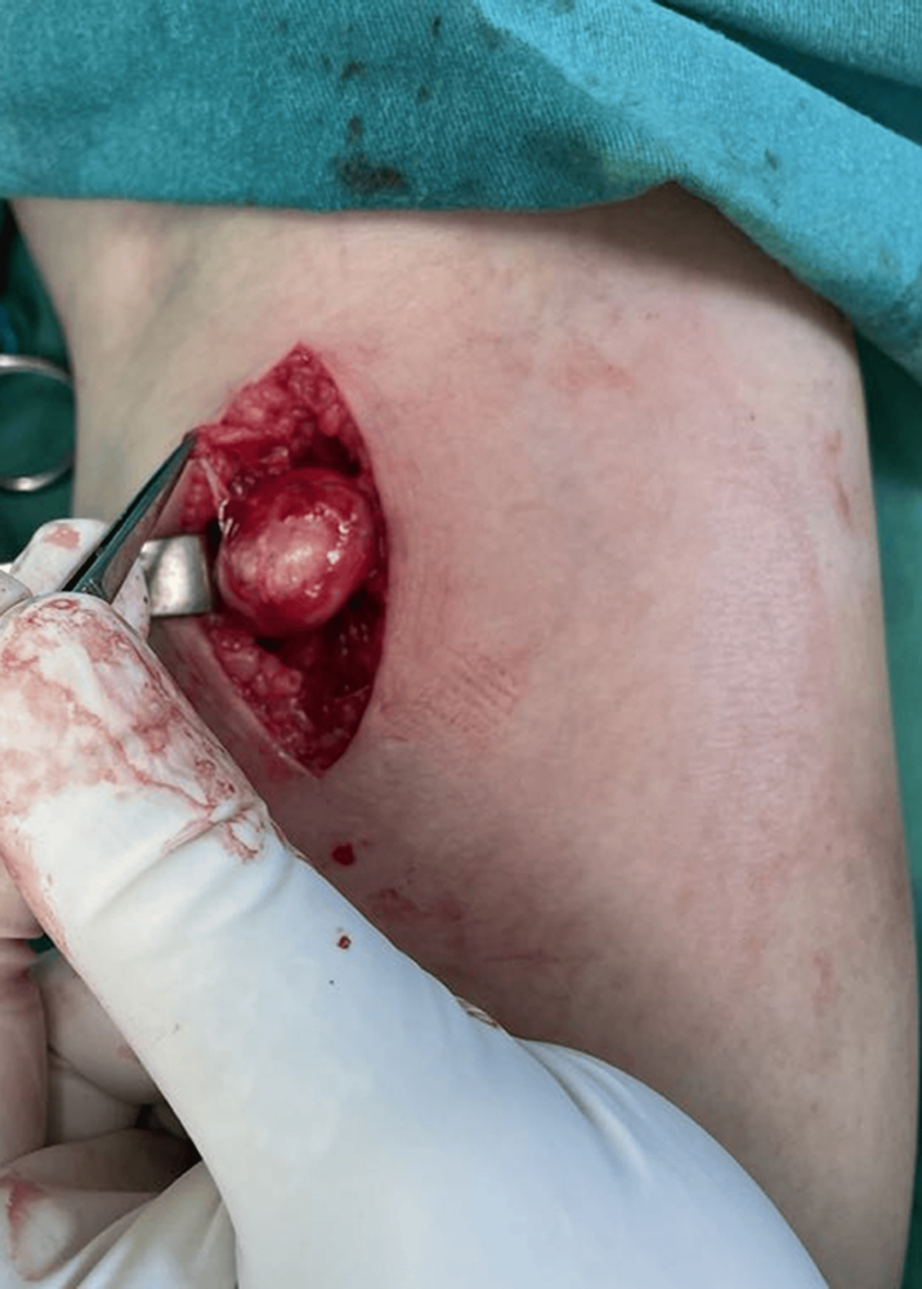
Intraoperative dissection of the sural nerve enveloping the tumor after enucleation of the tumor.

Based on these results, a benign neurogenic tumor, such as schwannoma, was strongly suspected, and the patient was referred for surgical treatment. The surgery was performed under spinal anesthesia. A longitudinal incision was made on the posteromedial side of the right leg, and the mass was dissected from the surrounding tissues. The tumor and the proximal and distal portions of the affected nerve were exposed, revealing an oval formation originating from the MSCN (Figure 3).

**Figure 4:**
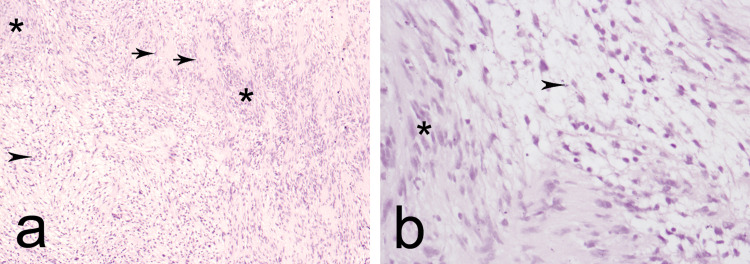
Hematoxylin and eosin staining at ×10 (a) and ×40 (b). Asterisk (*): Antoni A; black arrowhead: Antoni B; black arrow: Verocay bodies.

A longitudinal incision was made in the epineurium and the epineurial layers were gently peeled out until the shiny surface of the mass was exposed. The entire mass was subsequently shelled out as a single piece. Although the proximal part of the MSCN was partially damaged in the process, it did not require reconstruction. The intraoperative observations were consistent with the diagnosis of schwannoma, which was verified histologically. Elongated cells with defined edges and nuclei with dense chromatin, areas sparse in cellular material, and isolated foam cells with typical Antoni A and B regions were identified (Figure 4).

The patient’s postoperative course was uneventful. The patient had no evidence of tumor recurrence nor any neurological deficits at two months of follow-up.

## Discussion

We present a case of a 2 cm slow-growing (over two years) encapsulated schwannoma involving the MSCN. There was pain during the palpation of the mass. Paresthesia, local swelling, and tenderness were the main symptoms. SSs are rare findings [2], and few cases have been described in the literature, none of which involved the MSCN. El Ghazoui et al. described a schwannoma of the LSCN in the same area in a 28-year-old male [11]. The patient had paresthesias in the posterolateral aspects of the knee. Because of confusing symptoms and the small size of the tumor (0.5 cm on MRI), schwannomas may remain asymptomatic until the mass has compressed a subjacent neurovascular bundle [11]. Yamamoto et al. reported the case of a 42-year-old male with a greater than 30-year history of a localized, painless, 3 cm swelling in the posterolateral aspect of the distal left calf [2]. However, there was a negative Tinel’s sign. Albert et al. described two cases of SS. The first was a 26-year-old female with tenderness, pain, and weakness in the calf. In the beginning, peripheral vascular disease and deep venous thrombosis were suspected but ruled out with Doppler ultrasonography [15]. The second patient was a 22-year-old male with a palpable mass in the lateral aspect of the heel and tenderness [15]. Bilenki et al. presented the case of a 54-year-old female with left lateral foot and ankle pain. The reported SS was posterior to the lateral malleolus and was 1.5 cm in diameter [16]. Anesly et al. reviewed the case of a 39-year-old male with a mass in the lateral aspect of his right mid-calf, similar to our patient [3]. Angelini et al. summarized four cases of schwannomas in the foot. They presented the case of a 58-year-old female with a slow-growing painful mass near the lateral malleolus of the left foot with a four-year history [8]. As schwannomas are usually asymptomatic or present with misleading symptoms, the correct diagnosis is often delayed. The differential diagnosis includes neurofibromas, benign perivascular tumors such as angioleiomyomas, and malignant peripheral nerve sheath tumors [2,3]. In addition, vascular conditions such as varicosities, deep venous thrombosis, and aneurysms should be considered [6]. It is challenging to distinguish schwannomas from other possible differential diagnoses only by imaging, and a pathological diagnosis is necessary. In contrast to neurofibromas, which are sometimes encapsulated, schwannomas are entirely encapsulated. Neurofibromas affect the distal parts of the nerves, while schwannomas typically affect the proximal parts of the nerves or the spinal nerve roots [6].

The main diagnostic methods include physical examination and imaging diagnostics [2,11]. X-ray is used to rule out bone involvement. Nerve ultrasonography and MRI are the most frequently used modalities [13]. Ultrasonography has been used as a first-line imaging tool to evaluate and diagnose any superficial neoplasms of the extremities due to its low cost and satisfactory diagnostic value [17]. However, this method cannot differentiate between schwannoma and other types of tumors such as neurofibromas and ganglion cysts; therefore, in some cases, further diagnostic tools should be considered [17]. Lee and Yoon conducted a study on 152 patients diagnosed with a hand ganglion cyst based on clinical examination and high-resolution ultrasonography [18]. After a surgical mass excision and tissue biopsy, eight of these patients were finally diagnosed with a hand schwannoma [18]. This result can be explained by degenerative changes in schwannomas such as cyst formation, calcification, and hemorrhage which can cause variable sonographic features that make the diagnosis difficult [19]. On the other hand, neurofibromas and schwannomas are known to have similar sonographic findings. Several studies reported cases in which differentiation was possible, where an entering or exiting nerve in the mass was shown [20]. However, ultrasound imaging has proven to be a cost-effective and accessible tool for tumor localization and measurement. On MRI with contrast, schwannomas are isointense or hypointense masses relative to skeletal muscles on T1-weighted images and hyperintense masses on T2-weighted images. Schwannomas are often located near significant nerves [21]. On MRI, it is possible to see the nerve entering or exiting the mass. This characteristic indicates a neural origin of the tumor, which is a critical diagnostic feature [21]. The split fat sign refers to the appearance of fat surrounding the nerve sheath tumor, often seen in the limbs. It presents as a rim of fat around the mass on T1-weighted images, suggesting a peripheral nerve tumor [21]. On cross-sectional images, schwannomas may display a bundle of small ring-like structures representing the fascicles of the nerve. The fascicular sign is typically seen in peripheral nerve sheath tumors [21]. The target sign is characterized by a central area of low signal intensity with a surrounding area of high signal intensity on T2-weighted images. This pattern is suggestive of a schwannoma or neurofibroma [21]. When observed together, these imaging features can significantly suggest the presence of a schwannoma. EMG is used in cases of muscle weakness. When a malignant schwannoma is suspected, a positron emission tomography (PET) scan with fluorine-18 α-methyl tyrosine is used to distinguish benign and malignant tumor variants [22]. Ahmed et al. emphasized that benign schwannomas may have variable uptake of 18F-fluorodeoxyglucose, and it cannot be used in PET to define malignancy in such patients [22].

The treatment of choice is surgical intervention, i.e., enucleation [7]. Surgery is necessary only in symptomatic patients. Otherwise, conservative treatment is possible [14]. After verifying the nerve, a longitudinal approach through the perineurium and careful dissection of the formation is preferred. The tumor is then excised by enucleation. Intraoperative nerve stimulation differentiates between functional and non-functional nerve fascicles [23]. It helps preserve nerve function and minimize postoperative neurological deficits. Unlike neuromas found in neurofibromatosis, which require nerve resection, schwannomas usually do not infiltrate fascicles [11]. MRI follow-up is needed every few months. Recurrence is rare [11]; however, after operative intervention, neurological deficits or worsening of the symptoms of pain and paresthesias are sometimes found [7,24]. The incidence of neurological complications after surgical treatment for schwannomas of the limbs varies, and there is no consensus concerning predictive factors for complications [14]. Hirai et al. concluded that schwannomas of major motor nerves are more likely to have complications [14]. According to Kim et al., 1.5-76% of patients with significant nerve schwannomas may have neurological complications [7]. The development of neurological deficits in patients has been shown to have a strong association with preoperative biopsy and previously attempted resections. This suggests that these prior medical interventions may increase the risk of neurological complications [24].

## Conclusions

The present case features an unusual type of SS involving the MSCN. Precise physical examination and MRI are important for the correct diagnosis. Clinicians should consider SS as a possible diagnosis in patients with a slow-growing palpable mass in the posterior aspect of the knee and calf located posterior to the lateral malleolus. The present case expands the current literature as it demonstrates the difficulty in diagnosing and treating an unusual form of SS and illustrates the effectiveness of careful surgical management in preventing complications.
